# Cinnamon (*Cinnamomum zeylanicum*) Oil as a Potential Alternative to Antibiotics in Poultry

**DOI:** 10.3390/antibiotics9050210

**Published:** 2020-04-26

**Authors:** Mohamed E. Abd El-Hack, Mahmoud Alagawany, Abdel-Moneim E. Abdel-Moneim, Noureldeen G. Mohammed, Asmaa F. Khafaga, May Bin-Jumah, Sarah I. Othman, Ahmed A. Allam, Shaaban S. Elnesr

**Affiliations:** 1 Department of Poultry, Faculty of Agriculture, Zagazig University, Zagazig 44511, Egypt; 2 Biological Application Department, Nuclear Research Center, Atomic Energy Authority, Abu-Zaabal 13759, Egypt; aeabdelmoneim@gmail.com (A.-M.E.A.-M.); Noureldeen.goda@gmail.com (N.G.M.); 3 Department of Pathology, Faculty of Veterinary Medicine, Alexandria University, Edfina 22758, Egypt; Asmaa.Khafaga@alexu.edu.eg; 4 Biology Department, College of Science, Princess Nourah Bint Abdulrahman University, BO. Box 24428, Riyadh 11671, Saudi Arabia; may_binjumah@outlook.com (M.B.-J.); sialothman@pnu.edu.sa (S.I.O.); 5 Department of Zoology, Faculty of Science, Beni-Suef University, Beni-Suef 65211, Egypt; allam1081981@yahoo.com; 6 Department of Poultry Production, Faculty of Agriculture, Fayoum University, Fayoum 63514, Egypt; ssn00@fayoum.edu.eg

**Keywords:** cinnamon, hypocholesterolaemic impact, antibiotic alternative, essential oil, poultry

## Abstract

The removal of antibiotic growth promoters (AGPs) as feed additives in poultry nutrition from the market in many countries has compelled researchers to find unconventional and safe alternatives to AGPs. Probiotics, prebiotics, enzymes, organic acids, herbs, immune-stimulants and essential oils (EO) have been investigated as feed additives in poultry production. Cinnamon (*Cinnamomum zeylanicum*), one of the oldest medicinal plants and widely used around the world, can be used in poultry rations in the form of powder or essential oil. Essential oils produced from aromatic plants have become more interesting owing to their potential effects as hypocholesterolaemic agents, antioxidants, antimicrobials, antifungals and stimulants of digestive enzymes. The potential insecticidal and antimicrobial activities of EO against pathogens that cause spoilage in agriculture crops and human diseases might be attributed mainly to the high content of volatile components (mainly cinnamaldehyde, eugenol and carvacrol) in cinnamon oil. The present review focuses on the effects of cinnamon oil as a feed additive on poultry performance, carcass traits, meat quality, hypocholesterolaemic impact, antioxidant activity, immunity and microbiological aspects.

## 1. Introduction

**Figure 1:**
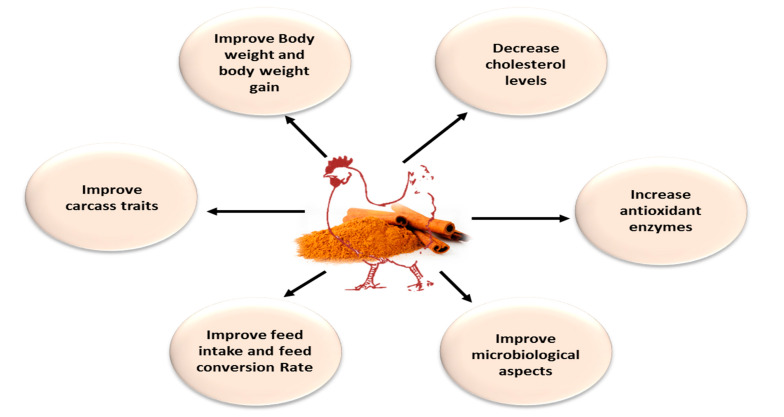
Advantages of dietary supplementation of cinnamon oil in poultry diet.

The poultry industry worldwide is one of the main agricultural subsectors [1]. The supplementation of poultry diets with natural products containing bioactive components has shown promising results [2,3,4,5,6]. The admission of essential oils (EO) extracted from aromatic plants in the formation of poultry rations could be a promising alternative to antibiotics that is safer for the environment. Essential oil extracts are widely used for skin care [7], aromatherapy, beauty treatments, herbal medicines and perfumery applications [8]. Aromatic plants and their essential oil extracts possess potential insecticidal activities and antimicrobial effects against pathogens [9,10,11,12], stimulating the effect on the digestive system [13]. These herbs and their extracts received more attention as possible antibiotic growth promoter (AGP) alternatives due to being natural, easily available, non-toxic and residue-free. This has makes them highly acceptable as natural feed additives for poultry (Figure 1).

Numerous plant extracts are used in the poultry industry as feed additives from these cinnamon essential oils (CEOs) and their components (cinnamaldehyde and eugenol) which possess antibacterial activity against *Parahemolyticus*, *Staphylococcus epidermis*, *Enterococus faecalis, Pseudomonas aeruginosa, Salmonella sp*., *Staphylococcus aureus* and *Escherichia coli* [14]. Moreover, cinnamon oil has strong hypocholesterolaemic, antioxidant, analgesic, antiulcer and anticandidial activities [15]. In addition, Wenk [16] demonstrated that plant extracts and herbs disrupt the growth of numerous pathogenic bacteria and stimulate the growth of beneficial bacteria in poultry digestive tracts.

## 2. Chemical Composition

Many studies were carried out to investigate the identification and quantification of the essential chemical components of volatile oils in the bark and leaf of wild and true cinnamon species. The concentration of some of the constituents identified in the cinnamon oil (leaf and bark) is presented in Table 1. The studies revealed that the chemical compositions of essential oil and oil yield were different as a result of many factors such as species, the part of the plant (leaf, bark, fruit and root) and the extraction method.

Abdelwahab et al. [21] indicated that the important components of *Cinnamomum altissimum* Kosterm bark oil were linalool (36.0%), terpinen-4-ol (6.4%), limonene (8.3%), methyl eugenol (12.8%) and α-terpineol (7.8%). The total phenolic components consisted of 50.41 μg GAE/mg of the oil. The antioxidant activity of the extract was 345.2 μM Fe^+2^/g dry mass using FRAP (ferric reducing antioxidant power) assay and with an IC50 value of 38.5 μg/mL using DPPH (1,1-diphenyl-2-picrylhydrazyl) assay. Liyanage et al. [22] revealed that the highest percentage of volatile component (3.23%) was found in *Cinnamomum verum* leaf oil. *Cinnamomum sinharajense* recorded the highest bark oil content (3.53%), while *Cinnamomum rivulorum* recorded the lowest stem bark oil (0.51%) and leaf oil (0.41%) contents. The essential oils of different cinnamon species contained fifteen very important volatile chemical constituents. *Cinnamomum verum* contained the highest percentage of cinnamaldehyde (67.57%) in comparison with the other wild cinnamon species. *Cinnamomum sinharajense* leaf recorded the highest content of euginol (87.53%). As Paranagama et al. [23] analyzed, the EO of the leaf, bark, fruit and root of cinnamon showed that the largest constituents of cinnamon fruit oil (CFO) were 8-caryophyllene (5.6%), T-cadinol (7.7%), and 6- and y-cadinene (36.0%). Approximately 84% of CFO is contained in sesquiterpenes, as the other parts of cinnamon comprise less than 9% of this group of components. Additionally, the main components of cinnamon leaf and bark oils were phenyl propanoids, but monoterpenes were the largest component (95%) of the root oil. Conversely, Kasim et al. [24] detected that the methodology of the extraction and the chemical components used were impacted by the cinnamon oil yield percentage. The highest oil yields were extracted by soxhlet extraction with dichloromethane (5.22%), hexane (3.84%) and petroleum ether (3.71%), respectively. However, using hydrodistillation methods recorded the lowest CEO yield extraction (only 1.82%). The results showed that nine major volatile compounds were obtained in the CEO extracted by hydrodistillation such as alcohols, ether, ester, aldehydes, ketone, alkenes and carboxylic acids. The highest percentage of trans-cinnamaldehyde 86.67%, which is considered to be the largest volatile component, was obtained by soxhlet extraction using hexane. Moreover, Atiphasaworn et al. [25] identified the volatile components of the EO of *Cinnamomum bejolghota* bark using GC-MS. Thirty six volatile components were identified with the largest constituents being borneol, γ-terpineol, terpinen-4-ol and 1,8-cineole. The EO of *Cinnamomum bejolghota* bark were firstly examined for their antifungal and antibacterial activities against gram-negative and gram-positive bacteria. The minimal inhibitory concentration (MIC) of *Cinnamomum bejolghota* bark oil against bacteria ranged from 31.25 to 62.50 μg/mL, and the inhibition against fungal pathogens (MIC = 125–500 μg/mL) was moderate. The powerful antimicrobial activity of *Cinnamomum bejolghota* bark oil was associated mainly with linalool, γ-terpineol, terpinen-4-ol, 1,8-cineole and borneol. Adinew [26] identified six components from the EO of cinnamon bark rising in Tepi (Southwest, Ethiopia). In addition, Şimşek et al. [27] analyzed the hydrodistillation of essential oil from the bark of *Cinnamomum zeylanicum* Lauraceae using Gas chromatography (GC) and Gas Chromatography Mass Spectrometry (GC-MS) systems. Twelve components comprising 99.2% of the cinnamon oil were identified. The largest components in this oil were cinnamaldehyde (88.2%), eugenol (1.0%) and benzyl alcohol (8.0%). Moreover, El-Baroty et al. [28] showed that essential oil extracted from the bark of *Cinnamomum zeylanicum* was characterized as being a unique, aromatic, monoterpene-rich natural source, with trans-cinnamaldehyde (45.62%) as the major constituent. The largest components were 3-phenyl, 2-propenal (87.013%), which comprised most of the components responsible for the therapeutic effect and cinnamon barks’ aroma.

## 3. Effects on Growth Performance

### 3.1. Body Weight and Body Weight Gain

**Figure 2:**
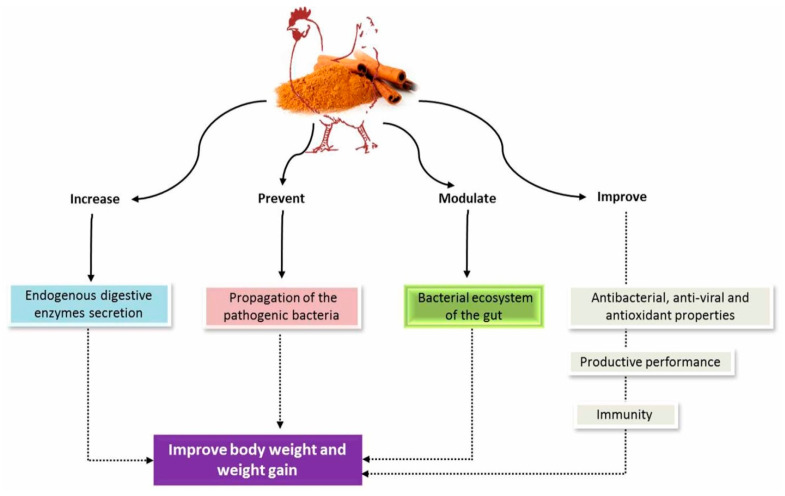
The effect of cinnamon oil on body weight and weight gain.

Many trials were carried out to assess the impacts of dietary supplementation with cinnamon (powder and oil) and CEO components as growth promoter agents. Al-Kassie [29] showed that broilers feeding on diet additives with CEO had a body weight gain significantly higher than the control (without CEO). In addition, Sarica et al. [30] illustrated that supplementation of CEO in quail diets had the same effects of antibiotic, probiotic, enzymes, mannanoligosaccharide, oregano essential oil (OEO), OEO plus CEO on body weight gain (BWG) of quail during the growth period of 0–35 days. Toghyani et al. [31] displayed that the addition of cinnamon to broiler diets by about 2 g/kg significantly improved the final body weight of the chicks. On the other hand, Mehdipour et al. [32] indicated that dietary supplementation with cinnamon oil (200 mg/kg) significantly increased the BWG of quails at 21–35 days old as a comparison with cinnamon powder, antibiotic (virginiamycin) and symbiotic. Shirzadegan [33] observed a significant increase in the final body weight of broiler chickens feeding on diets supplemented with different concentrations of cinnamon powder (especially at a level of 0.5%). Moreover, Devi et al. [34] showed that supplementation with a combination of CEO and ajwain essential oil in broiler diets significantly increased body weight at age 42 days. However, Lee et al. [35] stated that cinnamaldehyde supplementation in feed had no significant effect on female broilers’ weight gain, but water intake was decreased significantly. Muhl and Liebert [36] reported that the performance of broiler chicks was not significantly affected as a result of using commercial phytogenic feed additives that contain carvacol, capsicum oleoresin, cinnamaldehyde, chelerythrin and alkaloids sanguinarin. Koochaksaraie et al. [37] showed that the inclusion of cinnamon (0.5 to 2 g/kg diet) had no influence on the growth in broiler chickens. Moreover, Tonbak and Çiftçi [38] concluded that supplementation of cinnamon oil (*Cinnamomum zeylanicum* L.) in the diets at concentrations of 250 and 500 mg/kg diet had no significant influence on the live weight and live weight gain of the quail. Symeon et al. [39] added cinnamon oil to broiler diets at 0.5 or 1.0 mL per kg and summarized that cinnamon oil supplementation did not significantly affect broiler body weight at marketing age. In alternative strategies, emphasis was placed on preventing the spread of pathogenic bacteria and modifying the bacterial ecosystem of the intestine to improve the overall health and immune status, thereby improving productive performance. The enhancing effect of EO on the feed efficiency and growth performance was due to improving the immune system, regulating the gut micro flora, increasing endogenous digestive enzymes secretion and eliciting antioxidant, antibacterial and anti-viral properties [40,41,42,43]. The effect of cinnamon oil on body weight and weight gain is illustrated in Figure 2.

### 3.2. Feed Intake and Feed Conversion Rate

Studies related to the impact of cinnamon oil on feed intake (FI) and feed conversion rate (FCR) were contradictory, while many researchers [32,44,45] concluded that cinnamon oil has beneficial effects on FI and FCR. Al-Kassie [29] clarified that the chicks fed on diets containing 200 ppm EO resulting from a combination of thyme and cinnamon achieved significant increases in feed efficiency and FI compared to the control. Similarly, Ciftci et al. [46] suggested that broilers receiving a diet supplemented with 500 ppm cinnamon oil showed the best feed conversion efficiency in comparison with avilamycin (antibiotic) groups and the control. In addition, Mehdipour et al. [32] found that quails’ diet supplemented with cinnamon oil (200 mg/kg) significantly improved FCR compared to the control group (0–35 days), while FI was not affected. Moreover, Şimşek et al. [44] reported that the addition of cinnamon oil to the diets significantly reduced FCR. Torki et al. [45] indicated that FCR was significantly lessened in laying hens housed under cold stress conditions (8.8 ± 3 °C) and fed on the diets including Zn and CEO (combined or single) compared with those fed on the control diet. In addition, Mehdipour and Afsharmanesh [47] showed that the supplementation of cinnamon oil or virginiamycin to quail diets at a level of 200 mg/kg had the same significant beneficial effects on FCR compared to the control group at day 35; however, feed intake did not differ among the groups. In another study, Pathak et al. [48] demonstrated that enramycin supplementation (125 mg/kg feed), or a combination of calcium formate and cinnamaldehyde (500 mg/kg diet), to broilers orally challenged with *E.coli* (10^8^ bacteria/bird) on day 14, significantly improved FCR compared with the control group, and concluded that antibiotics can be replaced with EO and organic acid. Contradictory studies were reported by Symeon et al. [39], Sarica et al. [30], and Lee et al. [35] showing that cinnamon oil or powder did not significantly affect the FI or FCR of birds. Lee et al. [35] pointed out that cinnamaldehyde supplementation in feed had no significant influences on the FI and FCR of female broilers, however, water intake was significantly decreased. In addition, Hernandez et al. [49] indicated that broilers feeding on diets treated with 200 ppm essential oil extract (EOE) from cinnamon, pepper and oregano had no significant alterations in FI or FCR at 14 and 21 days of age. Moreover, Tonbak and Çiftçi [38] reported that supplementation of cinnamon oil (*Cinnamomum zeylanicum* L.) to the diets at concentrations of 250 and 500 mg/kg had no significant impacts on FCR of quail.

## 4. Carcass Traits

The potential impacts of CEO on carcass traits were studied by a number of investigators and the results consisted of beneficial effects or no effect of the essential oil. The effects of CEO on abdominal fat and fatty acid compositions of liver in broilers were studied by Dalkilic et al. [50] who indicated that saturated fatty acid (SFA) levels of broiler feeding on diets containing cinnamon oil at levels of 500 or 1000 ppm were lower, but polyunsaturated fatty acid, omega3 and fatty acid levels of the liver lipids were higher compared with the control (feeding basal diet) and antibiotic (avilamycin) groups. In contrast, Hernandez et al. [49] suggested that broilers feeding on diets treated with 200 ppm EOE had no differences for gizzard, proventriculus, pancreas, liver and intestine weights at 14 and 21 days of age. Moreover, Tonbak and Çiftçi [38] illustrated that supplementation of cinnamon oil (*Cinnamomum zeylanicum* L.) to the quail diets at concentrations of 250 and 500 mg/kg had no significant effects on carcass characteristics of quail. On the other hand, Symeon et al. [39] added cinnamon oil to broiler diets at 0.5 or 1.0 mL per kg and summarized that cinnamon oil supplementation did not significantly affect the carcass traits and the internal organs weights, although cold carcass weight was the highest in the cinnamon oil-supplemented group (0.5 mL/kg). As Devi et al. [34] suggested, no significant differences in broiler carcass traits were recorded as affected by the addition of cinnamon and ajwain essential oils at levels of 3 and 4 g/kg of feed, respectively. In addition, Gomathi et al. [51] reported that carcass characteristics such as ready-to-cook yield, abdominal fat, eviscerated, liver, gizzard, heart and giblet weights as a percentage of live body weight were not changed by the supplementation of cinnamon oil at levels of 250 or 500 mg/kg and coated sodium butyrate at 0.09% or 0.18% in broiler diets. In addition, the special aroma of cinnamon is desirable in poultry diets, and causes the elevation of the carcass content of unsaturated fatty acids and the alleviation of its content of SFA.

## 5. Blood Parameters

Most studies which investigated the effects of CEO on blood biochemical parameters illustrated the valuable impacts of CEO on these parameters, particularly its effects on lipid profile, antioxidant activity and immunity. Al-Kassie [29] and Ciftci et al. [52] reported that serum levels of catalase enzyme activities, glutathione peroxidase, ω-6 fatty acids, total unsaturated fatty acid ratio and blood phagocytic activity were increased significantly in broilers’ feeding diets that contained CEO. While serum malondialdehyde (MDA) level, cholesterol levels, total saturated fatty acid ratio and alanine aminotransferase (ALT) activity were decreased significantly in CEO groups compared to the control and antibiotic groups. Similar findings were reported by Yang et al. [53], who revealed that broilers feeding on diets mixed with different levels of CEO—alone (50, 100, 200, 400, or 800 mg of CEO/kg), or combined with bamboo leaf flavonoid (BLF) (100 mg CEO and 16.7 mg BLF/kg or 200 mg CEO/kg and 33.3 mg BLF/kg)—had significant impacts on liver MDA contents at 21 days old and serum IgM contents at 42 days old. Moreover, Torki et al. [45] concluded that the supplementation combination of CEO and Zn to the diets of laying hens reared under cold stress conditions significantly reduced the serum levels of triglycerides and glucose and increased the plasma content of Zn compared with those fed the control diet. Additionally, Sarica et al. [30] theorized that quail diets combined with the supplementation of OEO plus CEO or mannanoligosaccharide resulted in a reduction of plasma, total cholesterol and triglycerides levels compared with the basal diet. Moreover, Abudabos et al. [54] showed that plasma thiobarbituric acid reactive substances, total protein, and globulin were significantly increased in broiler chicks growing on *Clostridium perfringens* contaminated diets and supplemented with a mixture of cinnamaldehyde, anise, carvacol, thyme, yucca extract and oregano essential oils. Conversely, a study by Lee et al. [35] indicated that there was no change in plasma lipid concentrations caused by cinnamaldehyde supplementation in the feed. The reduction of cholesterol in the groups fed EO components may be due to its suppressing impact on 3-hydroxy-3-methylglutaryl coenzyme A reductase [55], which is a key enzyme in the synthesis of cholesterol [56]. On the other hand, active principles of spices such as eugenol, linalool and cuminaldehyde were shown to inhibit lipid peroxidation [57]. To summarize, the possible mechanism of EO in limiting and preventing cell membrane destruction by oxidative and production of free radicals, consequently reduces MDA formation.

## 6. Intestinal Microbiota

New strategies must be developed to boost poultry health [58]. The balance of intestinal microbiota (harmful and beneficial bacteria) is essential to get a healthy gut [59]. The potential effectiveness of CEO as a antimicrobial agent was studied by many researchers [47,53]. Most of the studies corroborated that CEO has a high level of antimicrobial activity and can be used as food biopreservative. Yang et al. [53] indicated that broiler diets supplemented with CEO (100 mg/kg feed) significantly decreased cecal *E. coli* relative multiplicity and significantly increased cecal *Lactobacillus* and *Bifidobacterium* relative multiplicity and concluded that CEO can be used as a potent alternative to antibiotics (aureomycin) as a way of improving the broilers’ cecal microbiota. Similar actions for CEO on quails were repeated by Mehdipour and Afsharmanesh [47], who showed that the ileal coliforms count was decreased and the ileal *Lactobacillus* count was increased in quails fed cinnamon oil (200 ppm/kg diet) compared with the control, antibiotic (virginiamycin) and cinnamon powder groups. In vitro, Gupta et al. [60] find that the inhibitory effect of CEO against bacteria and fungi was very alleviating and the lowest MIC was 1.25% (*v*/*v*) against *Klebsiella sp.*, *E.coli*, *Listeria monocytogenes* and *Bacillus sp.* and *Rhizomucor sp.* among the fungi. The antimicrobial potent agent for cinnamon oil was higher than cinnamon extract potency. In addition, it was concluded that the minimal bactericide concentration (MBC) and MIC for CEO were between 125–250 and 25–100 µg/mL, respectively. In contrast, it made no significant difference in ileal and cecal total bacterial counts, *Lactobacillus* and *E.coli* as a result of supplementation of a combination of calcium formate and cinnamaldehyde (500 mg/kg feed) to the broiler diets, however a significant decrease in *Salmonella* counts was obtained [48]. Cinnamon essential oil is mainly comprised of volatile components; the largest and most important contents are cinnamaldehyde followed by eugenol and carvacrol. These phenolic compounds manifest considerable antifungal and antimicrobial activity [61]. In addition, such volatile components as carvacrol manipulate the pH equilibrium of inorganic ions via their disruption of membrane integrity [62]. The antibacterial activity of cinnamaldehyde, eugenol and carvacrol was observed due to their preventive effects on pathogen microorganisms [49]. On the other hand, plant extracts and herbs disrupt the growth of numerous pathogenic bacteria and encourage the growth of beneficial bacteria in the digestive tracts of poultry [63]. Moreover, several in vitro studies demonstrated that herbal essential oils, such as cinnamaldehyde, thymol and carvacrol, exhibited strong antimicrobial effects against pathogenic bacteria including *Salmonella* and *E. coli* [64,65]. Chowdhury et al. [66] found that a supplemental 300 mg/kg of cinnamon bark oil (containing 505–977 g of cinnamaldehyde/kg) reduced the numbers of *E. coli* in the pre-cecal contents of broiler chickens. Certain research studies also indicated that the use of thymol with cinnamaldehyde would have the potential to prevent the proliferation of pathogens and contribute to better animal gut health [67,68]. Jamroz et al. [69] stated that the mixture of cinnamaldehyde, capsaicin and carvacrol decreased the count of *E. coli*, and improved the count of *Lactobacillus* in the intestinal tract of broilers. The positive effect of cinnamon oil on the population of *E. coli* might be due to the ability of this oil to disrupt the cell membranes of bacteria [68]. In addition, it has been reported that essential oil improves the release of mucus into the gut, which lowers the adhesion of pathogenic bacteria to the epithelium [70]. An in vitro study on ceacal content by Kollanoor-Johny et al. [71] found that the use of 20 mM of cinnamaldehyde, eugenol, thymol or carvacrol was sufficient in significantly reducing the concentrations of *Campylobacter* after 15 s of incubation. By 8 h of incubation, the use of 10 mM concentrations of these constituents was effective in reducing the count of *C. jejuni* by at least 5-log CFUs/mL [71]. Finally, the use of CEO as feed additives in poultry diets had some beneficial effects on the growth performance and lipid profile, antioxidant status, immunity and antibacterial activity of poultry as shown in Table 2.

## 7. Conclusions

The major findings of this study indicate that the supplementation of cinnamon essential oil extracts as feed additives in poultry diets have beneficial effects on the performance, hypocholesterolaemic, antioxidant activity, immunity and microbiological aspects. It is clear that cinnamon can be used as a potential alternative to antibiotics for more safety in the health, environmental and economic aspects of poultry industries.

## Figures and Tables

**Table 1:** The concentration of some of the constituents identified in the cinnamon oil (leaf and bark).

Compound	^1^ Concentration (%) in Cinnamon Leaf Oil	^2^ Concentration (%) in Cinnamon Bark Oil
Caryophyllene oxide	0.5	0.35
1,8-Cineole	0.6	1.02
Benzyl benzoate	3.0	0.01–0.37
Benzyl alcohol	0.2	0.14
Eugenol	74.9	0.39–2.37
Benzaldehyde	0.1	0.23–0.31
Camphene	0.3	0.08–0.12
Cinnamaldehyde	1.1	62.09–89.31
Cinnamyl acetate	1.8	1.48–2.44
Linalool	2.5	1.6–4.08
α-Pinene	1.2	0.37–0.50
β-Phellandrene	0.2	0.23–0.25
α-Cubebene	0.9	0.12–0.21
α-Humulene	0.6	0.01–0.28
Myrcene	0.1	0.05–0.40
Limonene	0.5	0.19–0.33
Cymene	0.8	0.02–1.31
β-Pinene	0.3	0.07–0.15
Delta-3-Carene	0.6	0.37
β-Caryophyllene	4.1	0.89–2.05
Phenylethyl alcohol	0.1	0.15
α-Terpinene	0.1	0.03
α-Phellandrene	0.9	0.01
α-Terpineol	0.3	0.01
α-Thujene	0.2	-
Safrole	1.3	-
Styrene	0.1	-
Elemene	-	0.08–0.33
Borneol	-	0.01–0.12
Coumarin	-	0.41–0.47
Benzenepropanal	-	0.41
Hinesol	-	0.36
T-cadinol	-	2.47
α-Muurolene	-	4.32
α-Amorphene	-	1.98

^1^ According to Schmidt et al. [17]; ^2^ According to Vazirian et al. [18], Kamaliroosta et al. [19] and Ainane et al. [20].

**Table 2:** Some effects of cinnamon essential oil on poultry.

Level	Bird Type	Age	Results	References
200 mg/kg	Broiler chicks	42 days	Improved body weight gain (BWG), feed conversion rate (FCR) and dressing% Decreased Abdominal fat% Decreased Cholesterol Improved blood haematology	Al-Kassie [29]
500 mg/kg	Broiler chicks	38 days	Increased glutathione peroxidase activity in the kidney and liver Reduced plasma malondialdehyde level and ALT activity Increased the phagocytic activity	Faix et al. [62]
500 mg/kg	Broiler chicks	35 days	Improved BWG and FCR No effects on carcass traits	Ciftci et al. [46]
1g/kg	Japanese quail	35 days	Decreased lipid profile	Sarica et al. [30]
1g/kg	Broiler chicks	35 days	Increased concentration of glutathione peroxidase and catalase Reduced level of malondialdehyde Lowered cholesterol levels of serum Decreased breast and thigh meat	Ciftci et al. [52]
200 mg/kg	Japanese quail	35 days	Improved BWG and FCR Increase water holding capacity of meat	Mehdipour et al. [32]
300 mg/kg	Broiler chicks	35 days	Improved the performance indices (BWG, FCR and performance index) Increased the carcass yield (dressed weight, drawn weight and eviscerated weight). Increased various hematobiochemical parameters	Gawande [72]
3 and 4 g/kg	Broiler chicks	42 days	Improved BWG and FCR No effects on carcass traits	Devi et al. [34]
200 mg/kg	Broiler chicks	35 days	Improved FCR Increased Lactobacillus and decreased Coliforms count in the intestine	Mehdipour and Afsharmanesh, [47]
250 mg/kg	Broiler chicks	35 days	Decreased meat cholesterol No effect on carcass characteristics, meat quality	Gomathi et al. [51]
400 mg/kg	Broiler chicks	42 days	Improved the immunity Decreased cecal *E. coli* Increased cecal Lactobacillus and Bifidobacterium	Yang et al. [53]
